# Integrins as a bridge between bacteria and cells: key targets for therapeutic wound healing

**DOI:** 10.1093/burnst/tkae022

**Published:** 2024-07-16

**Authors:** Dong Yu, Zhaoyu Lu, Yang Chong

**Affiliations:** Department of Traditional Chinese Medicine, The Affiliated Hospital of Yangzhou University, Yangzhou University, No. 368 Hanjiang Middle Road, Yangzhou 225000, Jiangsu, China; Department of General Surgery, The Affiliated Hospital of Yangzhou University, Yangzhou University, No. 368 Hanjiang Middle Road, Yangzhou 225000, Jiangsu, China; Department of Traditional Chinese Medicine, The Affiliated Hospital of Yangzhou University, Yangzhou University, No. 368 Hanjiang Middle Road, Yangzhou 225000, Jiangsu, China; Department of General Surgery, The Affiliated Hospital of Yangzhou University, Yangzhou University, No. 368 Hanjiang Middle Road, Yangzhou 225000, Jiangsu, China; Department of Traditional Chinese Medicine, The Affiliated Hospital of Yangzhou University, Yangzhou University, No. 368 Hanjiang Middle Road, Yangzhou 225000, Jiangsu, China; Department of General Surgery, The Affiliated Hospital of Yangzhou University, Yangzhou University, No. 368 Hanjiang Middle Road, Yangzhou 225000, Jiangsu, China

**Keywords:** Integrins, Wound healing, Wound chronicity, Bacterial infection, Extracellular adhesion molecules, Actin, Cytoskeleton, Inflammation

## Abstract

Integrins are heterodimers composed of α and β subunits that are bonded through non-covalent interactions. Integrins mediate the dynamic connection between extracellular adhesion molecules and the intracellular actin cytoskeleton. Integrins are present in various tissues and organs where these heterodimers participate in diverse physiological and pathological responses at the molecular level in living organisms. Wound healing is a crucial process in the recovery from traumatic diseases and comprises three overlapping phases: inflammation, proliferation and remodeling. Integrins are regulated during the entire wound healing process to enhance processes such as inflammation, angiogenesis and re-epithelialization. Prolonged inflammation may result in failure of wound healing, leading to conditions such as chronic wounds. Bacterial colonization of a wound is one of the primary causes of chronic wounds. Integrins facilitate the infectious effects of bacteria on the host organism, leading to chronic inflammation, bacterial colonization, and ultimately, the failure of wound healing. The present study investigated the role of integrins as bridges for bacteria–cell interactions during wound healing, evaluated the role of integrins as nodes for bacterial inhibition during chronic wound formation, and discussed the challenges and prospects of using integrins as therapeutic targets in wound healing.

Highlights

- A summary of the mechanism of action of integrins in the wound healing process is provided.
- Integrins and the mechanism of connectivity of different cells in wound healing are introduced.
- Mechanisms underlying the interaction of integrins within different species of bacterial cells are discussed.
- Advancements and prospects associated with integrin-targeted drug delivery for wound healing and bacterial infections are summarized.

## Background

**Figure 1:**
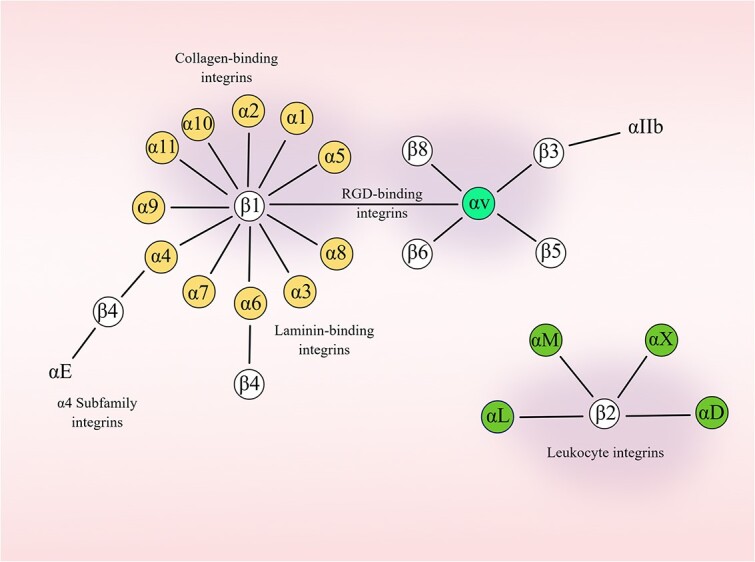
Members of the human integrin superfamily. A minimum of 18 α subunits and eight β subunits have been identified in humans, which together produce 24 different integrins. The integrin subunits that bind to each other to form heterodimers are connected using solid lines. Each integrin has a distinct ligand-binding specificity and tissue and cellular distribution. Reprinted with permission from Figdraw (www.figdraw.com)

Integrins are heterodimers composed of 18 α-subunits and 8 β-subunits. Integrins mediate the dynamic connections of extracellular adhesion molecules with the intracellular actin cytoskeleton and intermediate filaments. These connections are noncovalent in nature and serve to connect the extracellular matrix (ECM) to the cytoskeleton [1]. Cytoplasmic integrin-binding proteins regulate the binding of integrins to extracellular ligands and play a crucial role in integrin localization and transport. These proteins bind to the cytoplasmic tail of integrins and thereby mediate their connections to the cytoskeleton and signaling cascades that affect cell motility, growth and survival [2]. The targeting integrin sequence may be a simple tripeptide, such as Arg-Gly-Asp (RGD) or Leu-Asp-Val (LDV), or a more complex peptide such as GFOGER [3]. Integrins are classified into four types based on their binding properties. The integrins involved in leukocyte cell adhesion include α4β1, α9β1, αLβ2, αMβ2, αXβ2, αDβ2, α4β7 and αEβ7. Among these, the integrins α4β1, α4β7, α9β1 and αEβ7 recognize short specific LDV peptide sequences, which are also present in fibronectin. The most common integrin mediating leukocyte adhesion and migration is β2, which is characterized by sites in the ligand that are structurally similar to the LDV motifs [4]. RGD-binding integrins include αvβ1, αvβ3, αvβ5, αvβ6, αvβ8, α8β1, α5β1 and αIIbβ3, and are known to share RGD peptides with extracellular matrices (e.g. fibronectin, osteoadhesins, vitronectin and fibrinogen) via a common binding motif [5]. Collagen (GFOGER)-binding integrins, including α1β1, α2β1, α10β1 and α11β1, recognize the triple-helical GFOGER sequences in major collagens. Laminin-binding integrins, including α1β1, α2β1, α3β1, α6β1, α7β1, and α6β4. In addition, α10β1, α2β1 and α1β1, contain three αI structural domains and form a distinct laminin/collagen-binding subfamily [3,6]. In addition, these integrins exhibit different binding properties and varied tissue distribution (Figure 1 and Table 1) [7]. Integrins are involved in various physiological processes, including those related to trauma, immunity, infection, cell proliferation, inflammation, angiogenesis and tumors [8–11].

**Table 1:** Main secretory sites and functional roles of different types of integrins

**Type**		**Ligands**	**Secretion sites**	**Wound healing (granulation tissue)**
RGD binding integrins	αvβ1	TGF-β1, fibronectin, osteoblastin [7]	KC, EC	Modulation of the ECM Induces TGF-β activation [14] and mediates KC adhesion Mediates vascular remodeling [15]
	αvβ3	Fibronectin, FGF-2, TGF-β, vitronectin (hyaluronan), osteoblastin, platelet reactive protein, fibronectin [7,16]	EC, platelets, FB and macrophages	ECM regulation Angiogenesis: EC survival and FB proliferation [17,18] Inflammation: regulation of monocyte, macrophage and neutrophil migration [19,20]
	αvβ5	TGF-β, VEGF, osteoblastin, hyaluronan, platelet reactive protein [7]	EC, FB and skin KC	Fibrosis: induction of TGF-β activation mediates conversion of FB into myofibroblasts and matrix production [21,22]
	αvβ6	TGF-β1 and -β3, TGF-β, fibronectin, osteoblasts and metalloproteinases (ADAM)	KCs	Activation of TGF-β Inflammation/re-epithelialization: regulation of inflammation and KC proliferation [23,24]
	αvβ8	FN and TGF-β	Dendritic cells, FBs and ECs	Inflammation: may regulate inflammation by activating TGF-β [25]
	α8β1	TGF-β, tendonin, fibronectin, osteoblastin, hyaluronidase [7]	Myofibroblasts; vascular smooth muscle cells	Fibrosis [26]
	α5β1	Fibronectin, bone bridging protein, protofibronectin, thrombospondin, ADAM [27]	KC, EC, FB and leukocytes	Modulation of inflammation [6] Re-epithelialization: promotes KC migration [28]
	αIIbβ3	Fibrinogen, fibronectin, platelet reactive protein, hyaluronan, vascular hemophilic factor [7]	Platelets	Platelet activation and arterial thrombosis [29]
Leukocyte cell adhesion integrin	α4β1	Thrombospondin, VCAM-1, fibronectin, osteoblastin [30]	Leukocytes, FB	Regulation of inflammation Regulation of FB proliferation and differentiation [30]
	α9β1	EDA-FN, TN-C, ADAM, EMILIN1, and VEGF [31,32], bone bridging proteins, metalloproteinases-12 and 15	KC, FB, neutrophils and EC	Regulates KC and FB growth [33], neutrophil chemotaxis, and EC migration and angiogenesis [34–36]
	αLβ2	ICAM, JAM-1 intercellular adhesion molecule [ICAM]-1	Leukocytes	Mediates leukocyte extravasation through the endothelium [37]; enhances phagocytosis of bacteria by neutrophils [38]
	αMβ2	Fibrinogen, ICAM, heparin, FN, uPAR etc	Monocytes, macrophages, NK, neutrophils and T cells	Involved in leukocyte extravasation across the endothelium [37] Complexes with uPAR and its ligand uPA to promote fibrinolysis and clearance of fibrin clots from monocytes and neutrophils [39] Modulates inflammation and defends against microbial infection [38]
	αXβ2	Fibrous protein, ICAM, COL I [40]	Monocytes, macrophages, dendritic cells and NK	Involved in leukocyte extravasation [37] Regulates macrophages [41]
	αDβ2	ICAM-3, VCAM-1 and CCN1/Cyr6	Eosinophils	Involved in leukocyte extravasation [37] Modulation of inflammation and microbial infections [42]
	α4β7	VCAM	Leukocytes, dendritic cells	Involved in leukocyte extravasation
	αEβ7	Calcineurin	T lymphocytes, dendritic cells	
Collagen (GFOGER)/ laminin-binding integrins	α1β1	Laminin, collagen	EC, FB	Mediation of VEGF-driven angiogenesis, negative feedback regulation of collagen synthesis in FBL [43]
	α2β1	Laminin, collagen, thrombomodulin, e-calmodulin, tendonin	Platelets, KC, EC and FB, megakaryocytes	Mediates KC migration and VEGF-driven angiogenesis and keratinocyte adhesion [43,44]
	α10β1	Collagen type II	FB	May mediate FBL adhesion to collagen
Collagen (GFOGER) protein-binding integrins	α11β1	Collagen	FB	Mediates collagen gel reorganization, cell migration and proliferation [45]
Laminin-binding integrins	α3β1	Laminin Laminin-332 and laminin-511	Lungs, stomach, intestines, kidneys, bladder and skin	Mediates cell adhesion to the basement membrane and intercellular communication [46]
	α6β1	Laminin, platelet-derived growth factor	Platelets, EC, leukocytes and FB	May be involved in platelet–vessel wall interactions and angiogenesis [47], mediates keratinocyte migration [48], interaction with CCN1/Cyr61 promotes myofibroblast senescence and controls fibrogenesis [49]
	α7β1	Laminin	Cardiac and skeletal muscle	Mediates the binding of muscle fibers to the tendon junction [50] Vascular development and integrity [51].
	α6β4	LM-332	KC, EC	Promotes KC adhesion, migration and proliferation as well as EGFR signaling, regulates angiogenesis in the EC [52]

*FB* fibroblasts, *KC* keratin-forming cells, *EC* endothelial cells, *VEGF* vascular endothelial cell growth factor, *FN* fibronectin, *TGF-β* transforming growth factor beta, *EDA* extra domain A, *ADAM* a disintegrin and metalloproteinase, *CCN* Cyr61-CTGF-Nov, *Cyr6* cysteine-rich protein 6, *VCAM* vascular cell adhesion molecule, *uPAR* urokinase-type plasminogen activator receptor, *EMILIN1* elastic microfibril interface localization protein 1

**Figure 2:**
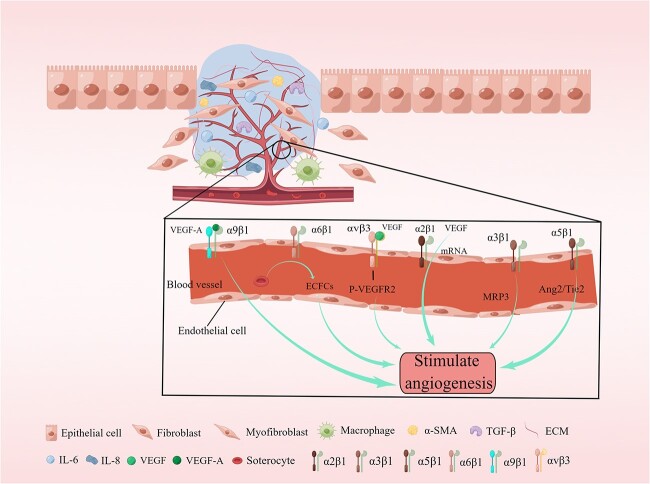
Stages of formation of new capillaries during wound healing. Vascular endothelial growth factor (VEGF) induces a 5- to 7-fold increase in the protein expression of two collagen receptors, namely, α1β1 and α2β1 integrins, on the surface of dermal microvascular endothelial cells (ECs) through the induction of mRNAs encoding α1 and α2 integrins subunits. The α5 integrin localizes to cell junctions and participates in the angiopoietin (Ang)/Tie2 signaling pathway to maintain vascular homeostasis. The αvβ3 integrin acts in synergy with VEGF to activate angiogenesis in ECs through VEGF receptor 2 (VEGFR2) phosphorylation. The α6β1 integrin appears to promote platelet mediated angiogenesis associated with endothelial colony forming cells (ECFCs). VEGF-A induces endothelial and cancer cell migration by directly binding to the α9β1 integrin. *ECM* extracellular matrix, *IL-6* interleukin 6, *TGF-β* transforming growth factor beta, *MRP3* multidrug resistance-associated protein 3, *α­SMA* α-Smooth Muscle Actin, *Tie2* Tyrosine kinase with Ig and EGF homology domains 2. Reprinted with permission from Figdraw (www.figdraw.com)

**Figure 3:**
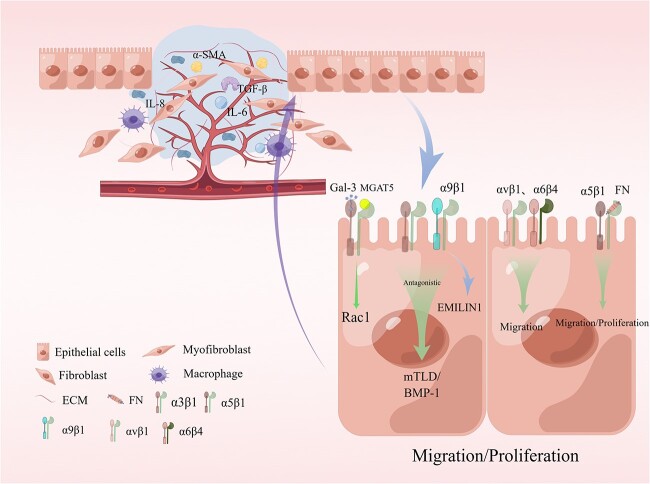
Stages of re-epithelialization during wound healing. Galectin-3 promotes epithelial cell migration by cross-linking with the MGAT5-modified complex N-glycans on α3β1 integrins and subsequently activating α3β1-integrin-Rac1 signaling to promote lamellar pseudopod formation. The interaction of α5β1 integrins with fibronectin (FN) contributes to keratinocyte proliferation in addition to promoting keratinocyte adhesion and motility on this matrix. The α9β1 integrin interacts with another extracellular matrix (ECM) component named elastic microfibril interface localization protein 1 (EMILIN1) to regulate keratinocyte proliferation, although the α9β1 integrin antagonizes the α3β1-dependent expression of mTLD/BMP-1 and skin basement membrane reorganization and maturation. The αvβ1 and α6β4 integrins also regulate keratinocyte migration. *IL-6* interleukin 6, *TGF-β* transforming growth factor beta, *MGAT5* mannosyl (Beta-1,6-) glycoprotein beta-1,6-N-acetyl-glucosaminyltransferase, *mTLD/BMP1* mammalian tolloid/bone morphogenetic protein 1, *Gal-3* galectin-3,* α-SMA* α-Smooth Muscle Actin. Reprinted with permission from Figdraw (www.figdraw.com)

Skin wounds heal successfully through various dynamic processes that occur in three overlapping phases—inflammation, proliferation and tissue remodeling [12]. Wound repair is tightly regulated by various factors, including cell–ECM interactions [12], growth factors and matrix metalloproteinases [13]. The integrin family of proteins regulates all aspects of the wound healing process, including hemostasis, inflammation, angiogenesis (Figure 2), re-epithelialization (Figure 3) and fibrosis (Table 1) [6,7,14–52]. Any disruption in these regulatory mechanisms at any of the three phases could result in chronic or non-healing wounds, and factors such as persistent inflammation and impaired barrier function [53,54], oxygenation response [55], bacterial infection [56], age [57] and disease state [53] could hinder effective wound repair. In real-life scenarios, chronic wounds are frequently accompanied by bacterial infections, with a few of these bacteria, such as *Staphylococcus aureus (*S. aureus*)* and *Pseudomonas aeruginosa (*P. aeruginosa*)*, capable of exploiting the integrin family to facilitate the development of chronic wounds, thereby perpetuating the presence of these wounds [56,58]. RGD-binding integrins, such as α5β1 and αvβ3, are the primary types of integrins identified to be mediating bacterial infection [59,60]. Therefore, the present review focuses on discussing the role of RGD-binding integrins as mediators in the bacteria–cell interactions occurring during the wound healing process. In addition, the potential of integrins as targets to impede bacterial involvement during wound chronicity is explored. Finally, the challenges and prospects of using integrins as targets in therapeutic wound healing are discussed.

## Review

### General mechanism of integrin attachment to the ECM

Integrins are the central mediators of cell–ECM and cell–cell adhesions. The cytoplasmic tails in the structure of integrins contain extracellular structural domains along with cytoskeletal and signaling proteins that bind to different components of the ECM. Such binding of integrins facilitates transmembrane signaling between the ECM and the cytoskeleton, allowing for the transmission of chemical and mechanical signals in and out of the cell [61]. The ECM in living organisms predominantly comprises a heterogeneous mixture. Moreover, cells express multiple isoforms of integrins, allowing for crosstalk among the different integrins and also between the integrin and non-integrin co-receptors [62]. Further detailed information on the general mechanisms through which integrins interact with the ECM may be obtained by referring to certain comprehensive reviews reported previously [6,63–65].

### Connection of RGD integrins to cells in wound healing

Platelets, neutrophils, macrophages, endothelial cells, fibroblasts and keratinocytes are the main cells involved in the wound-healing process.

### Connection of RGD integrins to platelets

Platelets contain three β1 integrins (α2β1, α5β1 and α6β1) and two β3 integrins (αvβ3 and αIIbβ3). These integrins promote platelet adhesion to ECM proteins, such as collagen, fibronectin and laminin, in addition to facilitating platelet aggregation [66].

In addition, αvβ3 facilitates cell adhesion and migration on immobilized ligands containing RGD motifs [67]. The widespread expression of αvβ3 could explain its lack of a distinct hemostatic function [66].

Expression of αIIbβ3 is reportedly limited to platelets [68], and this integrin is also the one present most abundantly on the platelet surface, where it facilitates both platelet adhesion and aggregation by binding to fibrinogen [69]. Platelet activation induces aggregation through interaction between integrin αIIbβ3 and plasma fibrinogen, which leads to hemostasis under pathological or physiological conditions [70,71]. The detailed role of platelet integrins in hemostasis and arterial thrombosis is thoroughly summarized in the report by Janus-Bell and Mangin [66].

Integrin α5β1 facilitates platelet adhesion, activation and aggregation on fibronectin and participates in thrombus growth [72]. The inhibition of α5β1 integrin was demonstrated to reduce soluble CD40 ligand (sCD40L)-mediated platelet activation [73].

### RGD integrins connect with inflammatory cells

Integrin αvβ3 is expressed in leukocytes, where it regulates the migration of monocytes, macrophages and neutrophils and the phagocytotic process in dendritic cells and macrophages, thereby modulating the progression of inflammation [19,20].

Integrin αvβ5 is expressed highly in mature intestinal macrophages. This integrin mediates the phagocytosis of apoptotic cells by macrophages and promotes tissue repair by regulating the homeostatic properties of intestinal macrophages [74]. Immature dendritic cells phagocytize apoptotic cells via αvβ5 and CD36 and cross-present antigens to cytotoxic T lymphocytes [75].

The expression of αvβ6 integrin is significantly elevated during tissue repair and malignancy [76]. The expression of integrin αvβ6 is correlated to the severity of inflammation, and integrin β6 deficiency significantly reduces the release of pro-inflammatory cytokines, such as TNF-α, interleukin 6 (IL-6) and IL-1β, in intestinal tissues with colitis. The inhibition of integrin αvβ6 expression was demonstrated to inhibit the polarization of macrophages toward the M1 phenotype while promoting macrophage polarization toward the M2 phenotype, thereby attenuating the dextran Sulfate Sodium (DSS)-induced inflammatory response in the mice with colitis [77].

The expression of integrin αvβ8 by human monocytes and macrophages activates transforming growth factor beta (TGF-β), a component that inhibits the production of pro-inflammatory cytokines and is disrupted in inflammatory bowel disease [78]. Studies have demonstrated that tumor cells expressing integrin αvβ8 evade host immunity by regulating TGF-β activation within immune cells [79].

As one of the fibronectin-binding integrins, α5β1 mediates the adhesion of neutrophils to fibronectin [80]. In addition, α5β1 regulates the adhesion behavior of neutrophils upon stimulation with various substances [81]. This is confirmed by the fact that during lung inflammation, α5β1 was demonstrated to be involved in the migration of polymorphonuclear leukocytes to the lungs [82].

### RGD integrins connect to endothelial cells

Integrins are the primary adhesion receptors that facilitate an interaction between endothelial cells and their extracellular microenvironment, thereby playing a crucial role in regulating the processes of cell proliferation, migration and survival [83]. The process of angiogenesis begins with the activation of endothelial cells, followed by the degradation of the vascular basement membrane and the sprouting of blood vessels within the mesenchymal stroma. In this process, the binding of the ECM to integrins provides vital signaling support for endothelial cell proliferation, survival and migration [84], thereby contributing to embryonic angiogenesis and adult angiogenesis [85,86]. Both *in vitro* and *in vivo* data indicate that numerous integrins, including α1β1, α2β1, α4β1, α5β1, α6β1, α6β4, α9β1, αvβ3 and αvβ5, present on endothelial cells regulate cell growth, survival and migration during angiogenesis [87].

The essential role of endothelial αvβ3 and αvβ5 integrins during angiogenesis was demonstrated through *in vitro* experiments involving a 3D assay conducted with bovine aortic and microvascular endothelial cells [88]. In addition, integrin αvβ3 acts in synergy with vascular endothelial growth factor (VEGF) to activate angiogenesis in endothelial cells by inducing VEGF receptor 2 (VEGFR-2) phosphorylation [89]. The interaction between integrins α5β1 and αvβ3 within endothelial cells having fibronectin is necessary for cell migration, and any disruption in this interaction impedes placental endothelial cell migration during fetal growth restriction [90]. Previous studies have demonstrated that focal adhesions of endothelial cells, which are concentrated on α5β1 integrin-selective substrates, efficiently recruit αvβ3 integrins [91]. In addition, catabolic inhibitors of αvβ3 integrin were demonstrated to impede VEGFR-2 signaling, thereby disrupting the angiogenic mechanisms, even in the presence of VEGF [92]. Therefore, drugs targeting integrin αvβ3 ligands enhance endothelial cell survival, implantation and angiogenesis [93].

ATN-161 is an antagonist of integrin α5β1, which induces apoptosis in neovascular endothelial cells, thereby effectively inhibiting ocular neovascularization [94]. The presence of soluble VEGF receptor-1 in the microenvironment of endothelial cells facilitates α5β1 integrin signaling, initiating cell migration and angiogenesis [95]. Moreover, the binding of Gln-362 in angiopoietin-2 to the α5β1 integrin facilitates the migration of both tumor and endothelial cells [96].

### RGD integrins connect to fibroblasts

Normal fibroblasts and granulosa fibroblasts, which are a combination of fibroblasts and myofibroblasts, express numerous integrins, including α1β1, α2β1, α3β1, α5β1, α11β1, αvβ1, αvβ3 and αvβ5. Both kinds of cells are capable of binding to a wide range of ECM molecules, such as collagens, fibronectin, other components of blood clots, CCN2/CTGF, etc. [97].

The αv integrins reportedly play a role in the differentiation of myofibroblasts, and the inhibition of αvβ5 or αvβ3 integrins was demonstrated to hinder the TGF-β1-induced differentiation of myofibroblasts in oral and dermal fibroblast cell cultures [98]. An increase in the expression of αvβ5 integrin induces α-Smooth Muscle Actin (α-SMA) expression in fibroblasts and enhances their activation by facilitating the recruitment of potential TGF-β complexes to the cell surface via the RGD motif. This activation then promotes their response to TGF-β1 [99]. Integrin αvβ5 also appears to regulate the function of α2β1 integrin in myofibroblasts and promote the maintenance of their myofibroblast phenotype [100].

Integrin αvβ3 reportedly hinders the infiltration of fibroblasts into wound clots through the inhibition of TGF-β1-mediated signaling [101]. However, the deficiency of αvβ3 integrin did not impact the differentiation of fibroblasts into myofibroblasts or wound contraction in a mouse model of cutaneous wound healing [101].

In the early stages of wound healing, an ECM protein named dermal bridging protein, which is present in the dermis, improves the adhesion of fibroblasts to the provisional matrix and facilitates the adhesion of these cells via α5β1 integrins [102]. For instance, syndecan-4 initiates the process of the endocytosis and trafficking of fibroblast α5β1 integrins, leading to the migration of cells toward the wound bed [103]. The presence of syndecan-C also facilitates the migration of fibroblasts into the granulation tissue via α5β1 integrin-dependent mechanisms [104].

### RGD integrin keratinocyte attachment

Epithelium reformation involves the collective migration of keratinocytes and the retention of certain cell–cell connections, although the precise mechanisms underlying their migration have not been definitively established [105,106]. While the key integrins for epithelial migration are α5β1 and α6β4 [107], several other types of integrins are also reportedly involved.

Injury may lead to upregulated expression of α2β1, α3β1 and α9β1 integrins, which subsequently translocate to the basal cell membrane of basal keratinocytes, where they induce αvβ1 and α5β1 integrins [108].

The interaction between α5β1 integrins and fibronectin may, in addition to enhancing keratinocyte adhesion and movement on this substrate, contribute to keratinocyte proliferation [109]. In contrast to α5β1 integrin, which is crucial for keratinocyte migration and fibronectin matrix assembly, αvβ1 integrin acts as a low-affinity fibronectin receptor that provides minimal support for keratinocyte migration [110].

As the potential binding integrin for fibronectin and myosin, αvβ6 integrin is induced in keratinocytes during the fusion of epithelial layers in wound healing [111]. Biopsies of the inner surface of the upper arm from adult volunteers during wound healing revealed that in the early stages of this process, basal expression of the epidermis appeared to include α5β1 and αvβ5 while αvβ6 was not present. In the middle stages, it was α5β1, αvβ5, and αvβ6. After the completion of the re-epithelialization process, α5β1 and αvβ6 were detected in all basal cells, while αvβ5 was not detected. Consequently, it was inferred that αv undergoes heterodimer binding, transitioning from β5 to β6 subunits during re-epithelialization [112]. In the later stages of wound healing, αvβ6 integrin expression is considerably upregulated in the basal lamina and multiple layers of basal lamina keratinocytes [111]. This upregulation could mediate TGF-β1 activation and regulate ECM deposition in the granulation tissue [113]. Enhanced keratinocyte integrin expression could potentially contribute to the development of chronic wounds. The expression of αvβ6 integrin is significantly upregulated in the epidermis during human chronic wounds, and its constitutive overexpression in mouse epidermis has been linked to the hyperactivation of TGF-β1 and increased vulnerability to chronic fibrotic ulcers in these animals [114].

In addition, keratinocytes interact with endothelial cells through integrins, and the removal of the α3 integrin subunit in the epidermis leads to compromised wound angiogenesis [17].

Wound healing is a dynamic process comprising three overlapping phases of inflammation, proliferation and tissue remodeling. The process of wound healing includes hemostasis in the initial stages of wound formation, followed by inflammation and granulation tissue formation during wound recovery (including angiogenesis) (Figure 2), and then re-epithelialization as the final stage of wound closure (Figure 3). These three processes involve various cell types, including platelets, inflammatory cells, endothelial cells, fibroblasts and keratinocytes. A review of the literature suggests that integrin-mediated processes are inherently inclined to facilitate their progression. Specifically, the activation of integrins promotes wound healing, and supplementation of appropriate integrins may further expedite the healing process. According to these findings, numerous directions and strategies for wound treatment could be developed. Conversely, blocking integrins might impede wound healing, a process that shares common characteristics with the perpetual wounds occurring in tumors.

### Integrins in bacterial infections

Prolonged inflammation may lead to non-healing wounds, such as chronic ulcers [115]. Chronic wounds may have complex causes, including local tissue hypoxia, wound bacterial colonization and repetitive ischemia–reperfusion injury, all of which contribute to wound development [116]. Inflammation resulting from the bacterial colonization of wounds remains one of the primary factors that delay wound healing [116]. The ECM is a non-cellular, 3D macromolecular network comprising collagen, proteoglycan/glycosaminoglycan, elastin, fibronectin, laminin and other glycoproteins. ECM regulates various cellular functions and plays a crucial role in maintaining normal body homeostasis [117]. ECM serves as the primary microenvironment for wound healing, and integrin-mediated adhesion to ECM is believed to play a crucial role in this process. Most chronic wounds at this stage are infected with bacteria, primarily Gram-positive bacteria such as *S. aureus* and *Staphylococcus hemolyticus*, although infection with Gram-negative bacteria such as *P. aeruginosa*, *Acinetobacter baumannii* and *Escherichia coli* are also reported [118–120]. The main mechanisms of these bacterial colonization organisms such as bacterial biofilm formation [121,122]. Biofilm formation is associated with the regulated synthesis of ECM components [123]. Biofilms comprise different bacterial species and contribute to the chronic nature of most wound-healing processes. Further, bacteria with biofilms exhibit high resistance to antibiotics [124].

### Integrins and *S. aureus*

*S. aureus* is a highly significant pathogen for humans and is recognized for its involvement in hospital-acquired infections and methicillin resistance. Accordingly, *S. aureus* is considered a global clinical concern [125]. *S. aureus* causes a range of surface and systemic diseases and has been commonly associated with oral mucositis. In addition, this bacterium plays a role in the development or exacerbation of several skin conditions, such as atopic dermatitis, carbuncles, cellulitis, boils, hair follicle infections, Kawasaki syndrome, impetigo, psoriasis and scalded skin syndrome [126–133]. *S. aureus* is considered the primary culprit for wound infections and is believed to impede the wound-healing process (Table 2) [17, 134–143]. A notable characteristic shared by almost all *S. aureus* isolates is the production of ECM-binding proteins, which are collectively referred to as microbial surface component recognition adhesion matrix molecules [144,145]. Microbial colonization of the host may occur when *S. aureus* attaches to ECM components, thereby initiating an infection [145]. For instance, cell wall-attached fibronectin-binding proteins A and B enable the bacteria to firmly adhere to the ECM protein fibronectin [146,147].

**Table 2:** Roles of different integrins during normal wound healing (granulation tissue) and bacterial infection

**Type**	**Granulation tissue**	**Bacterial infection**
α5β1	Regulates re-epitheslialization and promotes migration of keratin-forming cells [135]	Mediates the attachment of eukaryotic cells to the extracellular matrix protein fibronectin [136]
αvβ3	Regulates angiogenesis and promotes fibroblasts proliferation [17]	Mediates *Staphylococcus aureus* bloodstream infection [137]
αvβ6	Regulates inflammation and keratin-forming cell proliferation [138]	Regulates bacterial biofilms [139,140]
αIIbβ3	Mediates platelet aggregation [141]	Mediates adhesion of *Staphylococcus aureus* to platelets [142,143]

**Figure 4:**
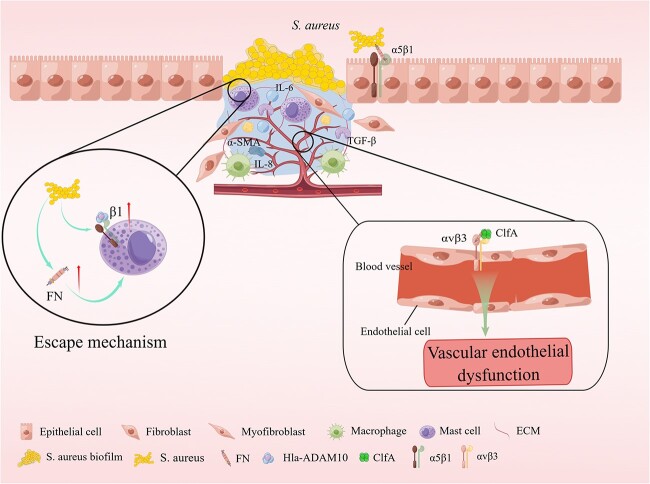
*S. aureus* evades the bactericidal mechanisms of the host cell. Fibronectin (FN) serves as a molecular bridge linking FN binding protein (FnBP)-expressing *S. aureus* to the α5β1 integrin present on the surface of human cells. FN tightly anchors *S. aureus* to its eukaryotic host cells and also facilitates the internalization of the microbe by human epithelial and endothelial cells and mouse fibroblasts. Further, the internalization of *S. aureus* by immortalized keratinocytes requires bacterial FnBPs and is mediated by the significant fibronectin-binding α5β1 integrin. *S. aureus* counteracts the extracellular bactericidal machinery of mast cells by increasing the expression of FnBP and inducing the Hla-ADAM10-mediated upregulation of β1 integrins in mast cells. Vascular endothelial dysfunction is attributed to the adherence of the *S. aureus* aggregation factor A (ClfA) to the αvβ3 integrins expressed on endothelial cells, with fibrinogen playing a key role during this process. The direct binding of the *S. aureus* surface protein iron-regulated surface determinant B iron-regulated surface determinant B (IsdB) to endothelial αvβ3 integrins plays a vital role in host cell adhesion and invasion, ultimately leading to a life-threatening disease. *IL-6* Interleukin 6, *ECM* extracellular matrix, *Hla-ADAM10* Hla-a disintegrin and metalloproteinase 10, *TGF-β* transforming growth factor beta, α-SMA α-Smooth Muscle Actin. Reprinted with permission from Figdraw (www.figdraw.com)

Integrin β1-containing receptors are recognized for their role in cell adhesion and their capability of cell attachment signal transduction to the ECM [148]. *S. aureus* is capable of invading eukaryotic cells by indirectly engaging the β1 integrin-containing host receptor. However, non-pathogenic *S. carnosus* is not invasive [149]. The α5β1 integrin is a cell surface receptor and a vital mediator of the attachment of eukaryotic cells to the ECM protein fibronectin [136]. Fibronectin has recently been demonstrated to serve as a molecular bridge linking fibronectin-binding proteins (FnBP)-expressing *S. aureus* to the α5β1 integrin present on the surface of human cells [150]. This interaction, besides allowing *S. aureus* to tightly anchor to its eukaryotic host cells, also promotes the internalization of the microbe by human epithelial and endothelial cells and mouse fibroblasts [151–154]. A study revealed that necrotizing soft tissue infections with *S. aureus* isolates had high rates of internalization and cytotoxicity to human myocytes, and the cellular basis of the high internalization rate in myocytes was the higher expression of α5β1 integrins in myocytes [155]. The ability of *S. aureus* to be internalized by and survive within the host cells, such as keratinocytes, might contribute to the development of persistent or chronic infections, eventually leading to deeper tissue infection or dissemination. The internalization of *S. aureus* by immortalized keratinocytes requires bacterial FnBPs and is mediated by the significant fibronectin-binding α5β1 integrin. However, unlike the internalization of immortalized keratinocytes, the internalization of *S. aureus* by native keratinocytes may occur via FnBP-dependent and non-dependent pathways [156]. Moreover, in oral infections, multi-strain oral biofilms inhibit the expression of αvβ6 integrin in gingival epithelial cells [140]. In addition, the periodontal inflammation caused by αvβ6 integrin deficiency reportedly results in a significantly altered oral microbiome [139]. However, the second fibronectin-binding integral protein αvβ6 on keratin-forming cells does not mediate *S. aureus* internalization [156] (Figure 4).

*S. aureus* counteracts the extracellular bactericidal mechanism of mast cells (MCs) by increasing the expression of FnBP and inducing Hla-a disintegrin and metalloproteinase 10 (Hla-ADAM10)-mediated upregulation of β1 integrins in MCs [157]. Interferon gamma (IFN-γ) intervention, partly by β1 integrins, drives the enhanced antimicrobial and pro-inflammatory responses of human MCs to *S. aureus* [158]. A study revealed that α-toxin interacts with β1-integrin, a receptor for host ECM protein, suggesting β1-integrin as a potential receptor for α-toxin on epithelial cells. This α-toxin reportedly inhibits *S. aureus* adhesion and internalization by interfering with integrin-mediated pathogen–host cell interactions [159].

In addition, an α5β1/αvβ3 integrin antagonist was reported to inhibit the invasion of epithelial cells by *S. aureus* [160]. In a study, vascular endothelial dysfunction was attributed to the ability of *S. aureus* aggregation factor A (ClfA) to adhere to the αvβ3 integrins expressed on endothelial cells, with fibrinogen playing a pivotal role in this process [60]. The direct binding of the *S. aureus* surface protein named the iron-regulated surface determinant B (IsdB) to the αvβ3 integrins within endothelial cells plays an essential role in host cell adhesion and invasion, leading to a life-threatening disease [137]. Therefore, αvβ3 integrin blockade appears to be an attractive target for the treatment of blood-borne infections caused by *S. aureus*. Further, a force-enhanced adhesion between IsdB and integrins could be one of the multiple mechanisms evolved by *Staphylococci* to effectively colonize or invade their hosts while resisting the shear forces encountered in various environments after infection [161]. *Staphylococcus. aureus* is capable of adhering to platelets via the high-affinity form of IsdB bound to the platelet integrin αIIbβ3 without the requirement of additional ECM proteins [142,143]. In addition, the αDβ2 integrins are reported to play a role in *Salmonella typhimurium* and *S. aureus* infections [162].

Integrin-linked kinases (ILKs) and Rac1 mediate the invasion of keratinocytes by *S. aureus*, and these bacteria may invade keratinocytes via the ILK-Rac1 pathway. Therefore, ILK could be a critical factor in preventing staphylococcal skin infections [163] and its use as a biological target in the treatment of *S. aureus* infections is speculated.

### Integrins and *P. aeruginosa*

*P. aeruginosa* is a ubiquitous Gram-negative bacterial species present in the environment. This species is responsible for serious infections in skin wounds, such as in patients with severe burns [164]. * P. aeruginosa* infections are difficult to treat because these bacteria are highly resistant to antibiotics and rapidly acquire resistance to new antibiotics [165]. This species is capable of forming biofilms [166] and is known to invade and multiply in the host cells. * P. aeruginosa* has been demonstrated to have the propensity to enter and colonize injured epithelial cells [167]. Substantial experimental evidence suggests that loss of epithelial polarity increases the harmful effects of *P. aeruginosa* on host cells [167]. The bacterium has evolved ways of manipulating host epithelial cell polarity to promote its infection [167,168]. Integrins usually remain confined to the basolateral plasma membrane of epithelial cells, and when *P. aeruginosa* reaches the basolateral side, it gains access to integrins [169]. The current studies on integrin-mediated *P. aeruginosa* infections are mostly limited to α5β1 and αvβ5 integrins in respiratory epithelial cells [170–172]. The *P. aeruginosa* lectin is a fucose-specific lectin named LecB, which clears integrins from the surface of cells at the wound margin and blocks cell migration and wound healing in a dose-dependent manner [169]. Further studies are, we need to determine the role of integrins during *P. aeruginosa* infections in infected wounds, which appears to be a suitable direction for the treatment of *P. aeruginosa* infections.

### Integrins and other bacteria

Integrins also mediate the infectious effects of a few other species of bacteria on their host organism. In the context of wound healing, no mechanistic studies to date have explored the interaction between integrins and *S. hemolyticus*. In addition, the current research on *A. baumannii* remains at the level of therapeutic applications to its infected organisms, such as a certain drug [173] or a therapeutic means for its treatment [174,175]. While no relevant research on the mechanism has been reported to date, this area appears to offer a broad scope for research.

Studies on the interaction between *E. coli* and integrins have focused mostly on intestinal infections [176] and urinary tract infections [177–179]. Hajj-Hussein *et al*. reported elevated levels of αvβ3 and αiiβ3 integrins in the treatment of colitis caused by *E. coli*, and this upregulation of integrins could be one of the reasons for increased inflammation and chronicity [176]. In addition, microRNA1976 is suggested to negatively regulate *E. coli*-induced vaginal inflammation in mice through the inhibition of CD105 and integrin αvβ6 expression [179]. Recently, studies on the role of *E. coli* and platelets revealed that the interaction of *E. coli* αIIbβ3 with Fc gamma Receptor IIA (FcγRIIA) activated platelets, which then causes thrombophilia or sepsis [180,181].

Entry into epithelial cells and prevention of primary immune responses are prerequisites for the successful colonization and subsequent infection of human hosts by *Streptococcus pyogenes* (group A streptococci). The interaction of group A streptococci with fibrinogen promotes the integrin-mediated internalization of bacteria into keratin-forming cells, and integrins α1β1 and α5β1 are the major keratin-forming cell receptors involved in this process [182]. Excessive bacterial invasion disrupts the attachment between the tooth surface and the epithelium, leading to periodontitis. Integrin α5 could be involved in the invasion of *Aggregatibacter actinomycetemcomitans* Y4 into gingival epithelial cells, and the resulting cascade of signal transduction decreases cell adhesion and reduces the defensive role of gingival epithelial cells by reducing integrin expression [183]. The adhesion of *Candida albicans* germ tube human endothelial cell lines is mediated by αvβ3, while this adhesion is significantly blocked by using either the anti-β3 monoclonal antibody GRGDSP peptide or heparin, and eliminated by using the two in combination [184]. Therefore, αvβ3 blockade could be used as a therapeutic tool against *C. albicans* infection. In addition, *Helicobacter pylori* induces the expression of integrin α5β1 and activates *H. pylori*-infected gastric epithelial cells via proteinase-activated receptor-2)-induced trypsin, and this process might have an important role in *H. pylori*-associated carcinogenesis [185].

Integrins serve as suitable receptors for bacteria invading eukaryotic cells, including endothelial and epithelial cells, while evading the bactericidal actions of cells such as MCs. Activation of integrins, which act as pivotal mediators between bacteria and host cells during infections, creates a favorable environment for bacterial invasion of the host cell, facilitating the internalization and colonization of bacteria. This phenomenon contributes to the inflammatory response elicited in the host organism during the initial stages of infection. In addition, heightened integrin responsiveness aids the invading bacteria in evading the bactericidal cells of the host, thereby perpetuating the persistence of the wound. Therefore, inhibiting the activation of integrins could be an effective strategy in the treatment of bacterial infections.

### Integrins and targeted therapy for bacterial infections

The integrin family is a large group of proteins in the human body that is involved in a variety of physiological processes. This family of proteins could be effectively utilized to regulate several pathophysiological processes in the organism. According to the mechanism of integrin-mediated bacterial infection in wound healing, it is speculated that bacterial infection in the vast majority of cases requires the regulation of integrins. Previously, it was believed that the interaction of staphylococcal alpha toxin with α5β1 integrin and the overproduction of tumor necrosis factor alpha (TNF-α) contribute to the destruction of epithelial cells during *S. aureus* infection [186]. Recently, *S. aureus* was demonstrated to counteract the extracellular bactericidal mechanism of mast cells by increasing the expressions of FnBPs and inducing Hla-ADAM10-mediated upregulation of β1 integrins in mast cells [157]. Therefore, inhibiting the targets associated with integrins could be an effective strategy for the treatment of *S. aureus* infections. Since the inhibition of the major integrin αVβ3 reduces the attachment of *S. aureus* to sheared human endothelial cells [60], blocking αVβ3 is considered an attractive strategy for the treatment of blood-borne infections caused by *S. aureus*. Alpha-melanocyte-stimulating hormone (α-MSH) is a neuropeptide produced primarily by the pituitary gland and also by several extra-pituitary cells, including skin keratin-forming cells. Evidence suggests that α-MSH exhibits anti-inflammatory and antimicrobial effects and reduces the internalization of *S. aureus.* In addition, α-MSH prematurely downregulates the production of integrins such as beta1 and heat shock surface protein 70 [187] to reduce the degree of infection and the inflammatory response.

In contrast, a study reported that in mice, *S. aureus* penetrated skin lacking ILK in the epidermis 35 times more than normal skin, indicating the great potential of ILK as a targeted therapy for the prevention of *S. aureus* skin infections [163]. Fibronectin or β1 integrin-blocking antibodies reportedly eliminate the IFN-γ-dependent *S. aureus* junctions, and IFN-γ is capable of inducing human mast cells under the mediation of β1 integrins to enhance antibacterial and pro-inflammatory responses to IFN-γ-dependent *S. aureus* [158]. The activation of integrins is perhaps more necessary in these cases. In addition, it is reported that *P. aeruginosa* produces the fucose-specific lectin LecB, which specifically removes the integrins from the surface of cells located at the wound margin and blocks cell migration and wound healing in a dose-dependent manner [123,169]. When appropriate, integrin supplementation could antagonize this blocking effect and promote wound healing.

In clinical trials for the treatment of sepsis, cilengitide prevented ClfA from binding αVβ3 located on endothelial cells, thereby decelerating infection without affecting the normal functioning of endothelial cells [60]. Therefore, targeted inhibition of αVβ3 treatment appears to be a locally applicable strategy for wound healing. The α5β1 integrin is one of the staphylococcal α-toxin receptors that mediate α-toxin cytotoxicity [186]. According to a study, α-MSH exerts a protective effect on the skin by reducing infection and inflammatory processes through the downregulation of β1 integrins [187]. LecB inhibitors could also be effective in the treatment strategy when used in combination with antibiotics [169,188]. In contrast, integrin receptors reportedly promote the increased binding of *S. aureus* to IFN γ-treated human Mast Cells (huMCs) [158], demonstrating the complexity of the MC response in the context of the cytokine environment. However, no relevant practical clinical studies have been conducted to date, and appropriate drug development and clinical trials are currently a top priority in the field of integrin-targeted therapy.

Therefore, under normal conditions without bacterial infection, supplementation of integrins is expected to promote wound healing. However, bacteria exploit integrins to invade the body and cause infections, turning integrins into accomplices. In this context, inhibiting integrins could be used as an effective approach to treat bacterial infections. The dual roles of promoting wound healing and inhibiting bacterial infection, however, raise a challenge in the treatment of chronic wound infections caused by bacteria, as integrins serve as a bridge between the bacteria and host cells. Therefore, developing a targeted drug specifically for integrins remains a challenge. For instance, in the case of *S. aureus*, supplementation of ILKs could potentially facilitate the penetration of the bacterium into the skin [163]. In the case of specific *Aureobacteria,* such as IFN-γ-dependent *S. aureus*, supplementation of β1 integrins could potentially enhance the bactericidal effect of the organism [158]. With regard to *P. aeruginosa*, supplementation of integrins counteracts the integrin-scavenging effect caused by the bacterium’s production of the fucose-specific lectin LecB, and under such circumstances, integrin supplementation would result in antimicrobial effects while also promoting wound healing, rendering it an advantageous approach. However, controlling the dynamic balance between the activation and inhibition of different fractional integrins during wound healing is key to wound healing and must be explored continuously.

## Conclusions

The integrin family is a group of functionally diverse protein families that play key roles in various physiological and pathological mechanisms by serving as protein–cell, cell–cell and bacterium–cell bridges. The integrins and their metabolites, in addition to regulating wound healing, play an important role in bridging the gap between bacterial infection and the host cell in the wound. Bacterial adaptation to the environment and gradual familiarization with the immune system could result in diminished local immune defenses against infections, thereby exacerbating the progression toward chronic wound states. Simultaneously, the body’s inherent healing mechanisms could become compromised, further impeding efficient wound healing. Therefore, a crucial initial endeavor to unravel the enigma of expediting chronic wound healing involves inducing an acute wound state. The involvement of the integrin family in mediating bacteria–host cell interactions during the intricate process of wound healing suggests the potential efficacy of incorporating selected integrin-related pharmaceutical agents or manipulating receptor expression to modulate integrin levels. Such interventions hold promise in regulating bacterial infections, thereby having the potential to interrupt the relentless progression toward chronic refractoriness, ultimately terminating the wound’s transition to a chronic state. Therefore, utilizing integrin-based approaches in wound therapy appears to be a feasible strategy. Currently, only a few studies have explored the strategy of activating integrins to block bacterial infections, and this area of research has a wide scope and warrants joint efforts to fill the existing gaps in research. *S. aureus* and *P. aeruginosa* are the two most common bacteria (Gram-positive and Gram-negative, respectively) detected in hospital-acquired infections. The two species have been discussed in the present review, with a focus on the mechanism of their invasion of the host organism via integrins, to provide a systematic review that will serve as a reference for advancing the treatment of clinical bacterial infections and a summary of recent studies on integrins and their related derivatives used as target therapeutics. In conclusion, the use of integrins as targets for blocking bacterial infections has great potential.
